# Influence of Bagging on the Development and Quality of Fruits

**DOI:** 10.3390/plants10020358

**Published:** 2021-02-13

**Authors:** Muhammad Moaaz Ali, Raheel Anwar, Ahmed F. Yousef, Binqi Li, Andrea Luvisi, Luigi De Bellis, Alessio Aprile, Faxing Chen

**Affiliations:** 1 College of Horticulture, Fujian Agriculture and Forestry University, Fuzhou 350002, China; muhammadmoaazali@yahoo.com (M.M.A.); ahmedfathy201161@yahoo.com (A.F.Y.); libinqi2020@126.com (B.L.); 2 Institute of Horticultural Sciences, University of Agriculture, Faisalabad, Punjab 38040, Pakistan; raheelanwar@uaf.edu.pk; 3 Department of Horticulture, College of Agriculture, University of Al-Azhar (branch Assiut), Assiut 71524, Egypt; 4 Department of Biological and Environmental Science and Technologies (DiSTeBA), University of Salento, Via Prov. le Lecce-Monteroni, 73100 Lecce, Italy; andrea.luvisi@unisalento.it (A.L.); alessio.aprile@unisalento.it (A.A.)

**Keywords:** fruit skin color, light-induced coloration, fruit appearance, bag material, anthocyanin content, texture

## Abstract

Fruit quality is certainly influenced by biotic and abiotic factors, and a main quality attribute is the external appearance of the fruit. Various possible agronomical approaches are able to regulate the fruit microenvironment and, consequently, improve fruit quality and market value. Among these, fruit bagging has recently become an integral part of fruits’ domestic and export markets in countries such as Japan, China, Korea Australia and the USA because it is a safe and eco-friendly technique to protect fruits from multiple stresses, preserving or improving the overall quality. Despite increasing global importance, the development of suitable bagging materials and, above all, their use in the field is quite laborious, so that serious efforts are required to enhance and standardize bagging material according to the need of the crops/fruits. This review provides information about the effects of bagging technique on the fruit aspect and texture, which are the main determinants of consumer choice.

## 1. Introduction

Fruits are a source of numerous compounds essential for the human body and are included in a well-balanced healthy diet. Although fruits and vegetables are low in calories, the nutritive value of fruits has gained interest nowadays, being the source of health-promoting vitamins, fibers, minerals [1,2], phytochemicals and bioactive compounds, which help to prevent cancers and cardiovascular risks [3,4,5]. Sufficient intake of fruits and vegetables replaces harmful saturated fats and sugars from the body and enhances healthy nutrients and dietary fiber [6]. The Food and Agriculture Organization of the United Nations (FAO) and the World Health Organization (WHO) recommend consuming at least 400 g of fresh fruits and vegetables each day [7]. However, physical and biochemical attributes of fruits are greatly influenced by environmental factors [8,9]. 

The bagging technique, which was first utilized in Japan in the 20th century for pears and grapes, is now widely applied in Asian countries (Japan, China, Korea), Australia and the USA, protecting fruits from the surrounding environment (mainly from light and pathogens, then stresses related to temperature, water/humidity, and air movement) with a sort of shield—a physical barrier around the fruit [9]. In fact, bagging consists essentially of enclosing a young fruit in a food bag by capping the bag with a ribbon or a clamp on the fruit stalk. Isolating the fruit from the external environment protects it during development from mechanical or biotic damage, especially in regions where fruits are prone to attacks by fungi, bacteria, insects and even birds [9]. The purpose is to obtain fruits without external imperfections, and with desired shape and color depending on the regional or national consumer preferences for the specific fruit. The expected color changes in comparison to non-bagged fruits can therefore correspond either to a reduction or an increase in color or even a greater homogeneity of the color itself. This is particularly important in markets, e.g., Japan, where aesthetic factors represent an important competitive factor. This review aims to show an analysis of the effects of bagging on the quality of the fruit, particularly on the effects on the external color, which is one of the main quality attributes of the fruit, taking for granted the protection provided by the bag against pathogens, pests or mechanical damages. 

## 2. The Role of Bagging on Fruit Quality

**Figure 1:**
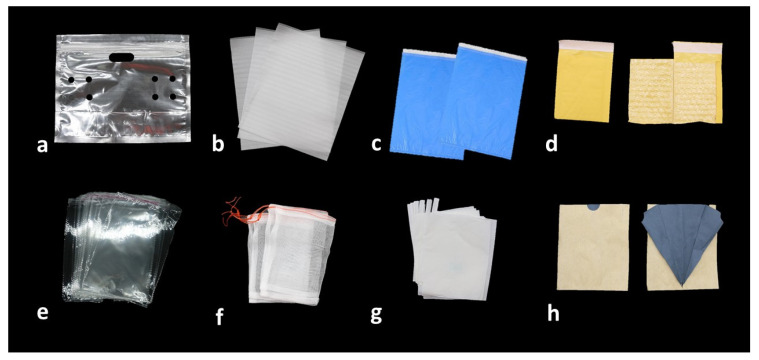
Different types of bagging materials used to improve the quality of fruits: (**a**) transparent paraffin bag; (**b**) nylon bags; (**c**) blue colored plastic bags; (**d**) two-layered bag (yellow paper outside and plastic inside); (**e**) cellophane bags; (**f**) organza bags; (**g**) paper bags; (**h**) two-layered paper bag (brown outside and grey inside).

Bagging technique is used specifically to enhance fruit appearance and quality, especially in Asia. There are different types of bags/bagging material (Figure 1). Initially, newspaper bags were used to wrap fruits to prevent damage from pests and diseases in Korea, but around 1985, artificially manufactured bags were introduced.

Though the bag production cost is high and the practice labor intensive, bagging with new materials has shown excellent results. A bag around a fruit controls sunlight, temperature, humidity, evaporation and mechanical damage. Bagging may also regulate harvesting time [10], and it can control pest attacks, especially fruit flies, minimizing residues of pesticides [11,12,13], which is particularly important during the rainy-season [14]. Thus, bagging is an excellent method to yield fruits with a very low input or residues of pesticide. In addition, bagging is able to promote the production of high-value organic fruits, as demonstrated for organic peaches in the southeastern United States by Allran et al. [15], who showed that fruit quality (size, Brix degree, acidity) was similar between bagged and control fruits, and by Campbell et al. [16], who reported that bagging protects against various pests and diseases but has minimal effects on organic peach quality. Similar findings were obtained by Araújo Neto et al. [17] after a bagging treatment of organic guava fruits. In addition, for organic fruits, bags can be doubled [18], or, in conventional farming, impregnated with insecticide [19] or sprayed with insecticides/fungicide before bagging [20].

Bagging can determine numerous changes in the physiology of the fruit and in the preservation of its characteristics, and particular attention has been paid to tropical fruits, for which there are numerous applications (Table 1), often found also for other types of fruits.

Yang et al. [33] proposed bagging as a very effective technique to modify the fruit microclimate, resulting in less fruit drop and reduced organic acid content in longan (*Dimocarpus longan* Lour.) fruits. The microenvironment inside the bag also showed a positive effect on the structure of apple peels [40] and reduced the cracking in longan [33] and date palm [37], and fruit sunburn and cracking in pomegranate [41]; in addition, the bagging of the banana bunch proved to be successful against chilling injury, preventing browning of the banana peel [19,42].

Bagging can increases fruit sugars and organic acid contents, two significant determinants of fruit organoleptic quality [43], although the response to bagging varies according to the fruits considered. Indeed, Zhou et al. [44] reported a decrease in sugar content after bagging of Chinese white olives (*Canarium album* (Lour.) Räusch.), as it was found for apple [45] and also date [38]. Conversely, Sarker et al. [21] and Islam et al. [46] reported an increase in sugar contents in bagged mango fruits, while Bently and Viveros [47] registered an improvement of fruit sweetness in Granny Smith apple. Huang et al. [48] stated that bagging has a non-significant effect on soluble sugars but decreases organic acids in pear fruits. Kim et al. [10] reported that peach fruits bagged with yellow paper (Figure 1d) showed an increase in total titratable acids due to low light, and white-colored bags determined an increase of soluble solid contents, chlorophyll and anthocyanins.

Xu et al. [31] investigated the effects of different light transmitting paper bags on fruits of two different cultivars of loquat (“Baiyu” and “Ninghaibai”); bagging materials included one layer white paper bags with ∼50% light transmittance (T_1_), and paper bags with a black inner layer and a grey outer layer with ∼0% light transmittance (T_2_). Fruit weight decreased, but fruit appearance improved with bagging, whereas total sugar content was higher in fruits subjected to T_1_ treatment than T_2_ and control. Both bagging materials reduced phenolics and flavonoids, with the lowest contents in T_2_ fruits [31]. Sharma et al. [49] reported that bag color also influences total fruit sugars in Red Delicious apples; Asrey et al. [50] indicated that red cellulosic bags applied 60 days after flowering are successful in producing high-quality pomegranate fruits (characterized by high consumer acceptability) in terms of total anthocyanin and ascorbic acid content, although with slightly lower calcium and total phenol; instead, Pantone^®^ 1205C bags determined a delay in pomegranate fruit development and ripening, which were outweighed by a reduced incidence of peel sunburn and higher antioxidant activity [51]. Yang et al. [33] observed that in longan fruits, sugar content was not significantly affected by bag type but resulted in an increase of fruit size and reduced cracking. 

In apple, bagging determined a better absorption of calcium by the fruits with a lower incidence of bitter pit in the cultivars “Red Fuji”, “Fuji Suprema”, “Imperial Gala” and “Gamhong” [52,53,54,55]. 

Bagging technique leads to the production of more attractive fruits due to fewer blemishes and visible marks [9], particularly in apple [47,49,56,57], pear [12,58,59,60,61], peach fruits [10,62], pomegranate [41], mango [21,22,23,24,25], carambola [27], guava [14,28], litchi [29,30], loquat [31,32], persimmon [34,35] and yuzu [36]. In addition, post-harvest losses are significantly reduced for mango [26].

However, some studies have also reported a negative impact of fruit bagging, for example reduced concentration of essential elements in mango [63]; smaller fruit size for loquat, pear, pomegranate and apple [9]; lower content in sugars and organic acids in apple [45]; ascorbate decline in pear [64]; and a reduced level of total carotenoids in peach [65].

## 3. Light and Fruit Flavonoids

Light is required for the photosynthetic process that provides the chemical energy needed for plant growth and productivity. Moreover, plant metabolism, gene expression and plant processes (e.g., movement of stomatal guard cells, abscission, mineral absorption, phototropism) are regulated or conditioned by light [66,67,68,69].

Concerning fruits, several researchers have proposed that solar radiation can induce changes in the flavonoid levels in terms of both quality and quantity [70,71,72]. Others have observed that light can elicit the expression of genes such as phenylalanine ammonia-lyase (*PAL*), chalcone synthase (*CHS*) or flavanone 3-hydroxylase (*F3H*), which are involved in the biosynthesis of flavonoids [73,74,75]. F3H catalyzes the stereospecific 3b-hydroxylation of (2S)-flavanones to the dihydroflavonols and is required for the biosynthesis of flavonols and anthocyanins [73,74], representing antioxidant compounds able to protect leaves from high light intensity and other stressful conditions [75].

In *Arabidopsis*, the *BANYLUS* (*BAN*) gene encodes anthocyanin reductase, which converts anthocyanidins to their corresponding 2,3-*cis*-flavan-3-ols on the pathway of condensed tannins; in fact, a mutation in the *BAN* gene leads to the accumulation of anthocyanins and a loss of condensed tannins in *Arabidopsis* seeds [76]. A correlation between the expression of the flavonoid pathway genes and the anthocyanin accumulation was demonstrated in bilberry ripening fruits [77]; in addition, the upper bilberry leaves exposed to direct sunlight showed an increase in the expression of flavonoid pathway genes and a higher concentration of anthocyanins, catechins and flavonols in comparison with lower shaded leaves [78]. These data support a protective role of flavonoids against excess solar radiation, and that high light conditions increase the accumulation of anthocyanins [73,79].

**Figure 2:**
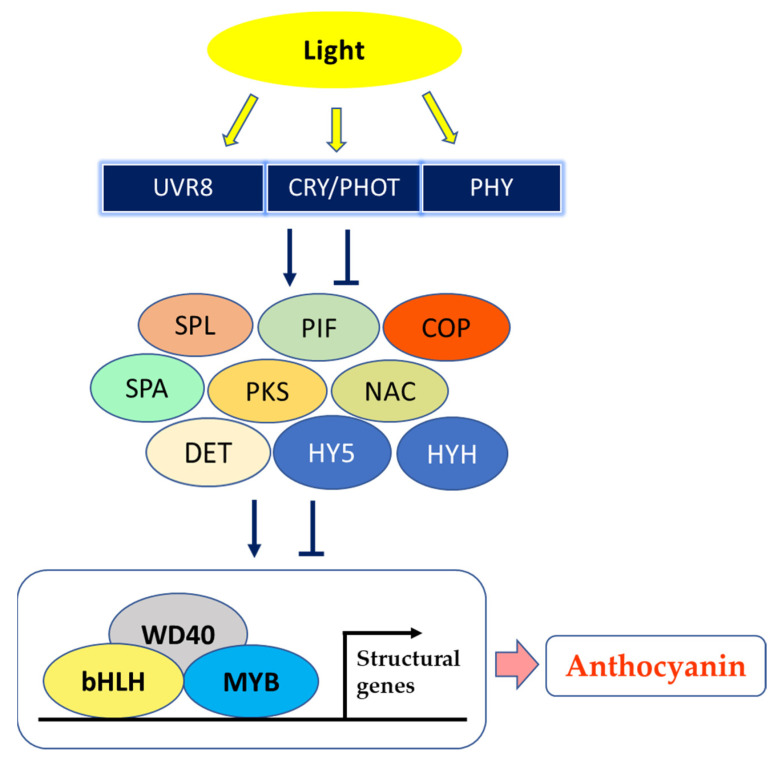
A model showing fruit peel coloration induced by light. UVR8—UV resistance locus 8; CRY—cryptochrome; PHOT—phototropin; PHY—phytochrome; SPL—SQUAMOSA promoter-binding protein-like; PIF—phytochrome-interacting basic helix–loop–helix transcription factors; COP—constitutive photomorphogenic; SPA—suppressor of PHYA; PKS—phytochrome kinase substrate; NAC—NAM (no apical meristem)/ATAF (Arabidopsis transcription activation factor)/CUC (cup-shaped cotyledon) transcription factor; DET—DE-ETIOLATED; HY5—elongated hypocotyl 5; HYH—HY5 homolog; WD40—WD40 domain-containing protein; bHLH—basic helix–loop–helix; MYB—myeloblastosis (modified after Chaves-Silva et al. [73], Zhao et al. [74], Ma et al. [81] and Zhou et al. [83]).

Interestingly, Zhao et al. [74] irradiated with UVA or UVB peach fruits, following 60–70 days of bagging, and proposed that UV light regulates the biosynthesis of anthocyanins, altering expression of several light receptors and in turn up-regulating several genes of the biosynthetic pathway; the working hypothesis was that photoreceptors interact with signal transduction elements of photomorphogenesis (constitutive photomorphogenic 1 (COP1), constitutive photomorphogenic 10 (COP10), phytochrome-interacting basic helix–loop–helix transcription factor (PIF), suppressor of phytochrome A (PHYA) (SPA), squamosa promoter-binding protein-like (SPL), which impact light-reaction effectors downstream (elongated hypocotyl 5 (HY5), elongated hypocotyl homologue 5 (HYH)) and the MYB–bHLH–WD40 (MBW) complex (myeloblastosis (MYB)/basic helix–loop–helix (bHLH)/WD40 domain-containing protein (WD40)) to regulate the transcription of the genes involved in the anthocyanin biosynthesis in response to light, as summarized in Figure 2 [73,80]. Especially, the “Granny Smith” apple underwent red pigmentation after bag removal, whereas both unbagged and bagged until harvest fruits did not acquire any tone of red; moreover, the expression of *PHYE*, *phototropin2* (*PHOT2*) and of the *UVB photoreceptors UV resistance locus8 (UVR8*), *DE-ETIOLATED* (*DET*), two *phytochrome kinase substrates* (*PKS1* and *PKS3*) and *COP1* tightly correlated with anthocyanin levels in apple skin [81].

Concerning the transcriptional regulation of anthocyanin biosynthesis, the most studied fruits are apple, strawberry and grape [73]. Particularly, in red-fleshed apple, two fruit variants have been identified; type I shows pigmentation in plant vegetative organs, and fruits exhibit a more intense color at early stages of development, reducing the color at ripening, whereas in type II apple pigmentations occurs only in fruit tissues (peel and flesh), which acquire color during maturation [82]. This means that light may regulate the biosynthesis of anthocyanins at different development stages in the two apple types. 

## 4. Bagging and the Color of Fruits

Since color is the main attractor for the purchase of fruit, bagging has been mainly used to obtain a specific color of the fruit skin and as a simple method to study the gene expression of the anthocyanin pathway in fruits [84]. Although some experiments have also been conducted on tropical fruits (as reported in the previous section), great attention has been paid to some pome fruit, stone fruit or vines.

In apple, besides the pigmented type I and type II varieties, other important commercial cultivars are typically acyanic, such as “Granny Smith” and “Golden Delicious”, but fruits turn to pink/red after bag removal [85]. The red coloration ten days after bag removal is more intense for “Granny Smith” than for “Golden Delicious; this was associated with a different level of *MdMYB1* gene expression, which seems to be the consequence of hypomethylation of the *MdMYB1* promoter in “Granny Smith” [85]. Further investigation analyzing differential expressed genes between unbagged, bagged and bag removed (before harvest) confirmed the importance of *MdMYB1* and other genes as *PHYE*, *PHOT2*, *UVR8*, *DET*, *PKS1*, *PKS3* and *COP1* for perception and transduction of the light signal after a dark period inside the bag [81]. From a practical point of view, the conclusion is the opportunity to realize the bagging of apples with materials that allow the passage of a substantial part of light radiation to maintain unaltered the color of red apples [86] and to avoid the blush of the skin in acyan apples [47,81]. Alternatively, bags must be removed a few weeks before harvest to avoid the red color reduction in cyan apples [57] (Table 2).

In pears the evolution of external coloration following bagging is similar to that of apple fruits, as summarized in Table 3; in fact, it was demonstrated that anthocyanin accumulates rapidly if the Red Chinese sand pear (*P. pyrifolia*) fruits are subjected to light within 10 days from bag removal [48]. Interestingly, the pigmentation patterns of *P. pyrifolia* (cultivar “Mantianhong”) differs from *P. communis* (cultivar “Cascade”) [88]; the first one develops a red color after bagging removal or postharvest UV/VIS irradiation. At the same time “Cascade” did not respond to light or UV exposure [88]. Additionally, the same authors indicated *PyMYB10* as the key regulator of anthocyanin biosynthesis in response to light [88]. Kim et al. [89] confirmed that in *P. communis* (cultivar “Kalle”), the anthocyanin contents in unbagged fruits remain higher than in bagged fruit. Qian et al. [90] employed bagging to study the light control of anthocyanin biosynthesis in pear fruit, demonstrating that miR156 was expressed in peels, increased after removing the bags, targeted four *SPL* genes and, additionally, PpSPL10 and PpSPL13 interact with PpMYB10. More recently Zhu et al. [91] have investigated the light-response patterns of 27 pear cultivars after bagging confirming that *MYB10*, *bHLH33* and *WD40* genes regulate the anthocyanin biosynthesis and that the expression of *HY5*, *PHYA*, *COP1*, *DET* and *PIF3* genes are also part of the color regulatory mechanisms in response to light. 

In peach, Zhou et al. [83] studied a red flesh variety showing that the color develops due to the expression of *PpMYB10.1*, which is activated by NAC transcription factors, in concert with the downregulation of the repressor *PpSPL1*. As with apples and pears, peach fruit bagging gives different results depending on the cultivar and the bag material [94]. The naturally deeply colored “Hujingmilu” peach and the light colored “Yulu” developed a deeper color when bagged with white non-woven polypropylene instead of yellow paper because the first type of envelope does not reduce the incoming of UV and blue light. The same authors proposed white non-woven polypropylene as a perfect replacement of yellow paper for peach bagging [94].

Later, Zhao et al. [74], still employing bagging on “Hujingmilu” and “Yulu” peach cultivars, demonstrated that both UVA and UVB induce pigmentation in “Hujingmilu”, while only UVB has an effect on “Yulu” fruits. In addition, Zhao et al. [74] supported the role of the light receptor as COP10 and HYH, and of the transcription factors belonging to gene families *MYB*, *bHLH*, *bZIP* and *NAC*, as summarized above. 

The intensity of the color tends to decrease in bagged fruit but, as for apples and pears, unbagging peach fruits ten days before harvest restores a blush comparable to the control [15]. Zhou et al. [95] indicated that shortening the bagging period increases the anthocyanin level in peach peel but reduces peel brightness and chlorophyll content. Additionally, the effects of bagging on carotenoid content were studied in yellow-fleshed peach [65], for which the use of yellow–black double-layered bags reduced the carotenoid level significantly (Table 4). 

The bagging treatments have low effects on grape berries because they inhibit anthocyanin accumulation in the skin and do not modify phenolic acid biosynthesis. A significantly elevated flavan-3-ol and flavonol syntheses were observed in re-exposed berries after early stages of bagging [96]. Moreover, bagging allowed for the detection of changes in the expression of *CRY2*, *HY5/HYHs* and *MYBA1* that matched with the biosynthesis of flavonoids in response to light [96]. A reduction of the color development and lower sugar contents in bagged grape berries was confirmed by Zha et al. [97] in “Shenhua” and “Shenfeng” cultivars, while fruit color and sugar content were rapidly restored by re-exposing the fruits to the light. Quite recently, Pisciotta et al. [98] reported that a bagging treatment is effective both in red and white table grapes; in fact, bagging led to a yield increase for the white varieties “Italia” and “Regal Seedless” and also for the red cultivar “Autumn Royal”, whose bunches, although of a slightly lighter skin color, showed increased color uniformity, reduced color variation and improved berry hardness. Furthermore, the bagging with white color non-woven polypropylene of “Muscat Hamburg”, which is a black berried grape, suitable both for wine-making and as table grape, determined a higher yield in terms of bunch weight, berry weight and wine yield [99]. 

Other results of grape bagging were a different production of volatile compounds and melatonin production. Ji et al. [100], investigating the influence of colored paper bags on the production of volatile compounds in “Kyoho” grape berries, indicated that the fruit bagging represents an effective technique to improve the grape aroma. Recently, Guo et al. [101] confirming that grape bagging delayed fruit coloring, sugar content, weight and ripening of the berries, and discovered that bagged berries of both “Cabernet Sauvignon” and “Carignan” cultivars synthesized more melatonin than did unbagged berries, suggesting a new interesting treatment in viticulture (Table 4).

Additionally, the bagging was recently employed to investigate the red blushed skin formation in apricot and kiwifruit (*Actinidia arguta*). Two blushed and two non-blushed apricot cultivars were compared; blush was not detected on the skin of bagged fruits, while transgenic experiments demonstrated the regulator role of PaMYB10 in apricot anthocyanin biosynthesis [102]. Bagging treatment on kiwifruit demonstrated that also in this fruit, light is necessary for normal skin coloration and that bagging suppression of anthocyanin biosynthesis occurs through inhibition of *AaMYB1* expression [103].

Finally, bagging screenings were employed to obtain non-photosensitive eggplants still able to produce an apparently average level of anthocyanins in the peel after bagging (with double-layer paper bags) treatment. These data allowed He et al. [104] to identify 22 transcription factors and 4 transduction elements as putative key regulators of the anthocyanin synthesis in the dark confirming bagging as a tool to study the fruit response to light.

## 5. Bagging and Fruit Microclimate 

Bagging results in changes in the microclimate around the fruit, as briefly mentioned in Section 2, but relatively few accurate measurements have been made because the main goal of the technique has always been to protect or improve the fruit’s appearance, avoiding a drop in texture, soluble solids and ascorbic acid. In addition, a lot depends on the material used for the bags, while the lack of standardization further complicates the comparison. In 2002, Amarante et al. [60] bagged pear fruits with micro-perforated polyethylene and determined that the temperature of the fruit skin with respect to unbagged fruit is higher on both the sunny side and the shady side during the growing season (a difference of 3 or 5 °C for the maximum skin temperature, respectively); the air temperature inside the bag was also higher, up to 5 degrees higher on a typical sunny summer day. Practically, the polyethylene bag gives origin to a mini greenhouse, influencing the cuticle deposition that is slightly less thick in bagged fruit and possibly reducing transpiration rates [60].

Further studies conducted in China were aimed at characterizing the temperature and humidity values in bagged fruits. Chen et al. [105] employed different types of bag on Actinidia, showing an increase of 0.7–0.9 °C for temperature and of 10.8–11.8% for relative humidity inside the bag. In the case of Fuji apples wrapped in two paper bags, the temperatures at the fruit surface varied in relation to the different fruit exposure on the canopy, and a reduction of 2.95 to 6.67 °C was observed between bagged and control fruits with identical exposure, while only minor differences were recorded for relative humidity between the inside and outside of the bags [106]. Cheng et al. measured the cucumber microenvironment employing four different types of bags, using two types of plastics and two types of papers of different colors [107]; they found only a minimal increase in temperature (less of 1 °C) both in sunny and cloudy days regardless of material and color, and a high increase in relative humidity (35–43%) for plastic bags in sunny days in relation to the higher transmittance of the plastic material (about 65–73%) compared to paper (23–35%); nevertheless, there were positive effects on fruit weight, morphology, nutritive quality and pesticide residues, and the color of fruit skin was lightened markedly in all four treatments, the cucumber color being linked to the chlorophyll level.

Concerning the higher relative humidity generated by bagging apples with two layer paper from 45 days after petal fall until 4 weeks before harvest, Hao et al. [40] observed a reduced accumulation of materials that form the cuticle in peel cells as a consequence of the higher relative humidity and that peel cracks increased after bag removal because of the re-exposure to sunlight and the lower relative humidity.

In peach bagged with polypropylene or one layer paper bag [43] the average temperature and relative humidity, recorded for 15 days before harvest, increased slightly in the bags, from 0.04% to 1.27% temperature and from 4.09% to 7.30% relative humidity; furthermore, an observed reduction in soluble solids was explained, indicating that the variation in temperature and relative humidity could affect the rates of transpiration and respiration. This conclusion is supported by a recent article of Morandi et al. [108], which indicated a positive effect of the high skin transpiration of bagged peach fruits in comparison to control fruits. 

Recently, air temperature was monitored inside paper bags that contain grape bunches [98]; the temperatures were slightly lower inside the bag in July, August and September (0.36, 0.23 and 0.15 °C, respectively) with a trend more relevant considering the cumulative temperatures inside the bag: −8.7, −5.4 and −3.7 °C in July, August and September, respectively, and +1 °C in October. Despite this, bagged bunches of all four cultivars tested achieved good quality, confirming the suitability of the bagging technique on grape.

Considering the effect of bagging on microclimate influencing biotic stresses, it is necessary to cite the work of Dai et al. [109], which confirmed that *Trichothecium* black spot is a disease associated with the fruit bagging and hypothesized that high relative humidity within bags is a key factor promoting *T. roseum* infection of bagged apple fruits.

Concluding, bagging reduces the light intensity on the fruit surface but at the same time increases temperature and relative humidity around the fruit, depending on the bag materials. However, the degree of positive or negative effects of fruit bagging strongly depend on the types of bag material and the bag covering techniques. Plastic determines a greater increase in temperature and humidity values than paper, but with wide variations in relation to the color and the presence of a double layer, which reduce the transmission of light and/or the accumulation of water vapor inside the bag.

## 6. Conclusions and Future Perspectives

Summarizing the effects of bagging on the red (anthocyanin) coloration of fruits, shading reduces color development in red flesh apple and peach, and in all red fruits such as, for example, grape (Table 2 and Table 4), while it does not modify the color of non-red fruits but rather favors a homogeneous coloration preventing the acquisition of an abnormal coloration such as greening of the skin in Asian pear [64]. On the contrary, since light/UV exposure stimulates color development, also in the orchard, the removal of the bag before ripening should allow the fruits to re-acquire red coloration, especially in the case of type II apples that normally develop coloration during maturation. The same phenomenon of induction of color also occurs in non-red apples [85] and peaches [74] after bag removal and UV treatment, which should be avoided in order not to modify the expected characteristics of the product. 

**Figure 3:**
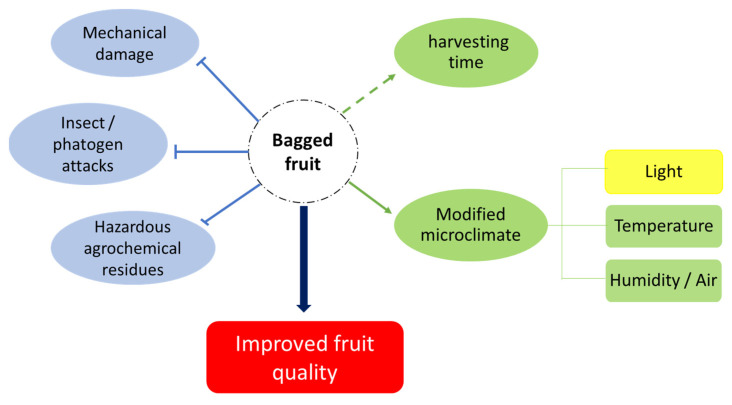
A model showing the influence of bagging on the quality of fruits.

Fruit bagging is a simple, safe and eco-friendly (adopting biodegradable bags) technique to protect fruits from pathogen and insect attacks and to improve fruit appearance and physicochemical properties (Figure 3). In fact, bagging treatments can improve the color of the fruit skin and make the final product more attractive than the “natural” untreated one. Anyway, the results vary from species to species and even between cultivars of the same species, so that it is necessary on the one hand to develop biodegradable bio-based materials with different levels of light transmittance suitable for the needs of different species or cultivars, and that researchers continue to study the bagging technique by applying standard protocols both for bagging time and for bag materials.

In particular, this technique seems to be able to find wide areas of application in the markets where the sale of organic products is developed, both in relation to the reduction of chemical inputs and the greater economic sustainability of this approach in a more profitable supply chain. In this regard, it is also hoped for the development of research aimed at the economic evaluation of these techniques, an area that is still almost completely unexplored but which, thanks to the availability of advanced analysis approaches (e.g., life cycle assessment analysis), could provide useful information to support the entire production chain.

## Figures and Tables

**Table 1:** The effects of bagging on color, quality and physiological disorders of some tropical fruits.

Crop/Cultivar	Bagging Start	Bagging Material	Effect	Ref.
Mango “Langra” and “Khirshapat”	30 d before harvest	black polybag, transparent polybag, brown paper	higher total soluble sugars and better physical quality of fruit	[21]
Mango “Nam Dok Mai #4”	48 d after full bloom	two-layered paper (brown outside and black inside)	improvement in fruit weight and skin appearance	[22]
Mango “Harumanis”	56 d before harvest	brown and black paper	improvement in skin color	[23]
Mango “Nam Dok Mai #4”	45 d after full bloom	low-density polyethylene	improvement in fruit weight and skin glossiness	[24]
Mango “Apple”	40–50 d before harvest	standard Kraft paper	reduction in lenticel discoloration	[25]
Mango “Khirsapat”	42 d before harvest	brown paper	reduced significantly post-harvest losses	[26]
Carambola “Malaysia”	10–31 d after full bloom	plastic, newspaper, and non-woven cloth	increase in fruit size, fruit weight and soluble solid content	[27]
Guava “Allahabad Safeda”	30 days after pollination	nylon nets, non-woven polypropylene, butter paper and brown paper	advanced fruit maturity, improved fruit weight, texture, visual appeal, quality and functional attributes	[14]
Guava “Tai-Kuo”	for 146 and 175 d during fruit development	waxed paper, nylon, Taiwan bag, telephone book paper	protection against pests and mechanical damage	[28]
Litchi “Feizixiao”	15 and 30 d after full bloom	cellophane or fabric	better fruit coloration/appearance	[29]
Litchi “Rose Scented”	14 d before harvest	perforated transparent polyethylene	reduction in fruit drop. increase in fruit size, higher soluble solids content	[30]
Loquat “Baiyu” and “Ninghaibai”	after fruit thinning (early April)	white paper (50% light transmittance) and two–layered paper (out grey, inside black—0% light transmittance)	improvement in appearance decrease in fruit weight	[31]
Loquat “Qingzhong”	after fruit thinning	paper	promotion in appearance, increased sucrose, glucose and soluble solids content, decreased fructose, sorbitol and titratable acidity content	[32]
Longan “Chuliang”	34 d after full bloom	perforated plastic, white or black adhesive-bonded	increased fruit size and fruit retention rate, reduced fruit cracking incidence	[33]
Persimmon “Shinsyu”	35–50 d before harvest	paper	no black stain	[34]
Persimmon “Fuyu”	1–4 months before harvest	white paper (40% shade)	reduced fruit blemishing (increase of blemishing with early removal)	[35]
Yuzu (*Citrus junos*)	early September	recycled Japanese phone book paper, grey colored paper and black polyester	significant reduction in fruit spot injury	[36]
Date Palm “Zaghloul”	at pollination time	transparent and blue polyethylene	reduction in tip cracked fruit	[37]
Date Palm “Succary” and “Khalas”	28 d after pollination	black, white blue, yellow plastic	acceleration fruit ripening	[38]
Date Palm “Helali”	30 d after pollination	black and blue polyethylene, paper	increased rate of fruit ripening	[39]

**Table 2:** The influence of bagging on physiological disorders, color and quality of apple fruits.

Apple Cultivar	Bagging Start	Bagging Material	Effect	Ref.
“Granny Smith”	40 d after full bloom (removed at 160 d after full bloom)	two-layer paper (outer brown, inner red)	increase in anthocyanin content after bag removal, increased expression of genes involved in light signal perception and transduction	[81]
“Qinguan” (deep-red cultivar), “Cripps Pink” (pale-red cultivar), and “Golden Delicious” (non-red cultivar)	45 d after full bloom	double layer paper (outer yellow, inner red paper coated with wax)	reduced anthocyanin accumulation in red cultivars, reduced sugar and organic acid contents	[45]
“Granny Smith”	114–118 d before harvest	brown paper	improvement of sweetness, sunburn reduction, 30 to 40% additional yield	[47]
“Delicious”	30 d before harvest	light yellow fabric	improvement in fruit color, firmness, and reduction in postharvest disorders	[49]
“Red Fuji”	40 d after full bloom	paper	better absorption of calcium in fruit	[52]
“Gamhong”	28–35 d after full bloom	Ca-coated paper	reduction in bitter pit	[53]
“Fuji Suprema”	40 d after full bloom	transparent micro-holed plastic and non-textured fabric	lower incidence of bitter pit, higher incidence of russeting, improvement in Ca content	[54]
“Imperial Gala”	40 d after full bloom	transparent micro-perforated plastic or non-textured fabric bags	reduction in bitter pit incidence	[55]
“Golden Delicious”	113 d before harvesting	two double layer paper: (a) outside grey–inside yellow; (b) outside newspaper–inside yellow	improved fruit skin, slightly decrease in size and weight	[56]
“Kurenainoyume”	39–54 days after full bloom (removed 29–48 d before harvesting)	light impermeable double-layered paper	incidence of cork spot in non-bagged fruits, no decrease in flesh firmness during storage	[57]
“Golden Delicious” and “Granny Smith”	40 d after full bloom (removed at 120 d or 160 d after full bloom)	two-layer paper (outer brown, inner red)	red/pink pigmentation after bag removal, more intense in "Granny Smith”	[85]
“Idared”	40 d after full bloom	1–3 layers of black hail net	small increase in mechanical properties Increase in russet susceptibility	[86]
“Fuji Raku Raku”	60–75 d after full bloom	double layer paper (outer grey, inner red)	lower internal browning with more rotting, lower phenolic content	[87]

**Table 3:** The influence of bagging on physiological disorders, color and quality of pear fruits.

Pear Cultivar/Species	Bagging Start	Bagging Material	Effect	Ref.
“Meirensu” and “Yunhongli No. 1" (*P. pyrifolia*)	20 d after full bloom (removed 1–3 weeks before harvest)	single- or two-layer paper with different levels of light reduction	improvement of anthocyanins accumulation removing bags 2–3 weeks before harvest	[48]
“Housui” (*P. pyrifolia*)	34 d and/or 83 d after full bloom	several colored paper combinations or transparent paraffin	improved fruit appearance (uniform, shine and smooth skin color with small lenticels)	[58]
“Carmen” (*P. communis*)	66 d before harvest (removed 13 d before harvesting)	paper bags: (1) white; (2) yellow; (3) black; (4) outside grey–inside yellow; (5) outside newspaper–inside yellow	red over-color formation removing bags before harvest, fruits were slightly smaller, improved quality of the skin	[59]
“Conference” (*P. communis*)	30 d after full bloom	micro-perforated polyethylene	reduction in skin blemish and russet	[60]
“Cuiguan” (*P. pyrifolia*)	20 d (changing the bag at day 45) or 35 d after full bloom	paper	fruits bagged earlier were brighter, with less russet, fewer dots and stone cells	[61]
“Cuiguan” *(P. pyrifolia)*	20 d after full bloom	double-layer paper (yellow outside, red inside)	ascorbate decline	[64]
“Mantianhong” (*P. pyrifolia*) and “Cascade” (*P. communis*)	20 d after full bloom (removed 10 d before harvest)	double layers of yellow–black paper	red skin coloration in response to light/UV irradiation	[88]
“Kalle” (*P. communis*)	20 d after full bloom	white, yellow and double layered black paper	reduced skin color intensity, best performance with white bags	[89]
“Meirensu” (*P. pyrifolia*)	40 d after full bloom (removed 10 d before harvest)	double-layered yellow–black paper	anthocyanin accumulation and expression of miR156 and its target *PpSPL* genes,	[90]
27 different cultivars (*P. pyrifolia, P. communis, P. bretschneideri, P. ussuriensis*)	40 d after full bloom, harvest 10 d before commercial maturity, then treatment with artificial light	double-layered paper (outer layer yellow outside and black inside, inner layer red)	increasing levels of anthocyanin under artificial light conditions.	[91]
“Chili” (*P. bretschneideri*)	77 d after full bloom	polyethylene and non-woven fabric	prevention of scald with non-woven fabric, higher scald with polyethylene	[92]
“Pingguo” (*P. bretschneideri*)	40 d after full bloom (removed 9 or 2 d before or at harvesting time)	paper	anthocyanin increase and up-regulation of *MYB* genes at day 9 after bag removal	[93]

**Table 4:** The influence of bagging on color and quality of peach and grape fruits.

Crop/Cultivar	Bagging Start	Bagging Material	Effect	Ref.
Peach “Hujingmilu” and “Yulu”	42 days after full bloom	yellow paper	UV-light induction of anthocyanin biosynthesis	[74]
Peach “Janghowon Hwangdo”	after final thinning (early June)	coated white paper, coated yellow paper, white paper, yellow paper and newspaper	improvement in the appearance and in the accumulation of anthocyanins	[10]
Peach “Hakuho”	before pit hardening, and 15 days before harvest	orange paper or orange triple and single parchment paper, 15%, 50%, 80% transmittance	decrease of the color intensity proportionally to the light reduction. Increase in aroma volatile content.	[62]
Peach “3D-8” and “C18”	50 d after full bloom, harvest at 70, 80 and 90 d after full bloom	double-layer paper (yellow outside and black inside)	reduced content in total carotenoids, low quality	[65]
Peach “Hujingmilu” and “Yulu”	96–100 days after full bloom, harvest at commercial maturity or 106-139 days after full bloom	yellow paper, and black, white, blue and grey nonwoven polypropylene bags	non-woven polypropylene bags determined the highest anthocyanin content in peel.	[94]
Peach “Hujingmilu”	50 days after flowering, bags removed at 90 or 105 days	paper single-layer, yellow	a short bagging period improves and stabilizes peel anthocyanin content reducing peel brightness and chlorophyll	[95]
Grape “Cabernet Sauvignon”	3 weeks after full bloom (with different timing) to harvest	two-layer paper (yellow outside, black coated with wax inside), with a bent straw	limited effects on berry quality positive correlation of phenolics to different light regimes	[96]
Grape “Shenhua” and “Shenfeng”	45 days after full bloom	white (light 25%) or shading light bags (light 0%)	incomplete color development, lower content of soluble sugar	[97]
Grape “Italia”, “Autumn Royal”, and “Regal Seedless”	berries at pea size (bagged at least 90 days)	paper	increased yield for the three cultivars and increased berry hardness for “Autumn Royal”, and “Regal Seedless”	[98]
Grape “Muscat Hamburg”	after fruit set	non-woven UV stabilized polypropylene of different colors	improved yield (both in summer and winter season)	[99]
Grape “Kyoho” (*V. vinifera* × *V. labrusca*)	5 weeks after full bloom	white, green, blue and red paper	promotion of accumulation of esters, inhibition of synthesis of aldehydes, alcohols, terpenes, ketones and acids	[100]
Grape “Cabernet Sauvignon” and “Carignan”	from fruit set	fruit bags with a black double-layer inside	promotion of melatonin biosynthesis in berry skins, delayed fruit coloring and ripening	[101]
